# Hidradeonoma Papilliferum of the Anus: A Case Report About the Relationship Between Neoplasms of the Mammary-Like-Glands and Hormones

**DOI:** 10.7759/cureus.13061

**Published:** 2021-02-01

**Authors:** Elizabeth J Olecki, Jeffery S Scow

**Affiliations:** 1 General Surgery, Penn State Health Milton S. Hershey Medical Center, Hershey, USA; 2 Colorectal Surgery, Penn State Health Milton S. Hershey Medical Center, Hershey, USA

**Keywords:** hidradenoma papilliferum, anal, mammary-like anogenital glands, neoplasm

## Abstract

Hidradenoma papilliferum (HP) is a benign pathologic finding that has been described primarily in the vulvar region. While thought to arise from ectopic tissue along the mammary line, it is now known to also arise from mammary-like anogenital glands (MLG), which are part of the normal anogenital cellular and glandular milieu. Previous work has demonstrated the relationship between HP and hormone receptors, but this has not been documented in a clinical setting. In this case, we present HP in a patient undergoing infertility treatments, presenting with a painful, enlarging anal mass. Upon histopathologic review, the mass was found to be an HP with no malignant changes. This unique case adds to the existing literature on perianal HP. While this case follows the known clinical and histological patterns of HP, the unique temporal relationship to in vitro fertilization (IVF) treatment supports previous in vitrowork on the relationship between HP and hormone receptors.

## Introduction

Hidradenoma papilliferum (HP) is a benign pathologic condition that has been primarily associated with the vulvar region. Originally thought to arise from ectopic or residual mammary tissue along the mammary line, it is now known to also arise from mammary-like anogenital glands (MLG), which are part of the normal anogenital cellular and glandular milieu. Histologically, they resemble breast tissue, but they do not arise from the embryologic mammary line [1]. Benign and malignant neoplasia of MLG, similar to the benign and malignant neoplasia of the breast, has been previously described. Because of the histologic similarities between MLG and breast tissue, some authors have suggested looking at these lesions “through the eyes of a breast pathologist” [2]. HP, histologically, is very similar to intraductal papilloma: papillae formed by branching tubules with fibrous bands between them [1]. Additionally, 90% of HP express estrogen receptors [3]. Despite occurring almost exclusively in females, no relationship between estrogen and HP has been documented in a clinical case.

HP occurs primarily in middle-aged Caucasian females. The typical age of the patients ranges from 25 to 66 years with the average age for vulvar and anal lesions reported to be 39 and 45 years respectively [4]. Clinically, these lesions are usually less than 2 cm in diameter and present as a painless, solitary, freely mobile mass with no overlying skin changes [5].

There have been case reports of ectopic MLG tumors on the eyelids, head, neck, axilla, and arms [6] as well as the perianal region. While MLGs are said to arise from “anogenital glands”, very few cases are associated with the anus. Over 90% of MLGs are located between the labia minora or majoria [2]. Less than 20 cases of anal HP have been reported in the literature [7].

While typically described as painless, we discuss a case that presented with the primary complaint of anal pain and a progressively enlarging anal mass in the setting of ongoing treatment for infertility.

## Case presentation

A 35-year-old female of Indian descent presented to our outpatient colorectal surgery clinic; she reported an enlarging perianal mass for the past three months. The mass eventually stabilized at approximately 1 cm. The patient reported that the mass had become painful, causing severe discomfort with sitting and bowel movements. She denied blood per rectum or changes in bowel habits.

The patient's past medical history was significant for endometriosis requiring multiple surgeries including diagnostic laparoscopy, myomectomy, laparoscopic ovarian cyst excision, and unilateral salpingectomy. Notably, the patient was currently undergoing injection in preparation for in vitro fertilization (IVF) to treat her infertility. IVF treatment had started prior to enlargement of the anal mass. She denied tobacco, alcohol, and drug use. She had no family history of colorectal or anal cancer or inflammatory bowel disease. 

Her physical exam was notable for a left-sided, perianal, fleshy, 1-cm mass, which was pedunculated on a short, narrow stalk. A digital rectal exam and anoscopy demonstrated no additional lesions and no enlargement of hemorrhoids. The lesion was non-tender and mobile with no overlying skin changes. At the time of initial examination, the lesion was suspected to be an enlarged lymph node, sebaceous cyst, or hypertrophied papilla.

Excisional biopsy was performed with local anesthesia. Examination after excision revealed that the mass was filled with a keratin-like material similar to a sebaceous cyst. The specimen was sent for pathologic review. 

After histologic examination, the lesion was reported to be an HP (papillary hidradenoma) with no atypical or malignant changes. In this case, no estrogen receptor immunohistochemistry was performed as the pathologic diagnosis was made without additional staining. The patient recovered well after the excision and was advised that she may proceed with her planned IVF treatment.

## Discussion

HP is a rare pathologic finding, with anal HP being even rarer. Diagnosis is rarely made clinically and is almost exclusively done by histopathology. HPs are benign lesions that are essentially cured with resection [8]. There are very low rates of recurrence once they have been completely excised. The malignant potential of HP is a controversial subject; even though some cases have been reported, some authors have speculated that these have been histologic misinterpretations [9].

A unique aspect of this case was the temporal relationship to the patient's IVF treatment. There are documented estrogen receptors on HP [2], with previous research by Offidani and Campanati finding that 90% of HP expressed estrogen receptors. These authors have suggested that HP might be controlled by ovarian steroid hormones [3]. This case exemplifies a close temporal relationship between IVF treatment and the growth of HP, lending support to this hypothesis. The clinical relationship between hormone stimulation and HP growth has not been previously documented. If HP is influenced by estrogen levels, this would be a newly found risk factor for the enlargement and growth of HP. 

Our patient was undergoing injections for planned IVF treatment. While many specific pharmacologic interventions exist for IVF, the goal is to recreate the natural midcycle luteinizing hormone surge in order to stimulate the ovaries and induce multiple follicles to mature for harvest [10]. Iatrogenic ovarian stimulation in IVF results in supraphysiologic estradiol levels, which are produced from aromatase in the granulosa cells of the follicle [11,12]. Unfortunately, we did not have a precise timeline as to when the patient had received IVF treatments and regarding the growth of her perianal lesion; however, we knew that the growth of the lesion had occurred after the initiation of IVF treatment.

This case illustrates the importance of employing a wide differential when approaching anal and perianal masses as well as the important role of histologic examination. In this particular case, the mass was suspected to be a number of things other than HP. The true diagnosis was only established after histologic review. This is not unexpected, as the diagnosis of HP is almost never made clinically [9]. We recommend that all excisions of perianal lesions be sent for pathologic evaluation in order to verify the diagnosis.

## Conclusions

In conclusion, this unique case adds to the existing literature on HP in the perianal region. While this case follows the known clinical and histological patterns of HP, the unique temporal relationship to the patient's IVF treatment supports the previous in vitro work on the relationship between HP and hormone receptors. Further research is needed to investigate more about the clinical relationship and determine the association between HP and hormones.
